# A feasibility study of sequenced TMS and TBS dosing in adolescents with major depressive disorder

**DOI:** 10.1016/j.transm.2025.100093

**Published:** 2025-04-08

**Authors:** Cicek N. Bakir, Paul A. Nakonezny, Dicle Buyuktaskin, Lucero Sangster-Carrasco, Irem Azamet, Jennifer Vande Voort, Paul E. Croarkin

**Affiliations:** a Mayo Clinic School of Graduate Medical Education, Mayo Clinic College of Medicine and Science, Rochester, MN, USA; b Department of Population and Data Sciences, UT Southwestern Medical Center, Dallas, TX, USA; c Department of Psychiatry and Psychology, Mayo Clinic Children’s Research Center, and Mayo Clinic Depression Center, Mayo Clinic, Rochester, MN, USA

**Keywords:** Transcranial magnetic stimulation, Theta burst stimulation, Intracortical facilitation, Biomarker, Adolescents

## Abstract

This feasibility study focused on a staged approach to transcranial magnetic stimulation (TMS) interventions for adolescents with depression. Adolescents (N = 6) who did not respond to standard TMS were offered a two-week course of either continuous or intermittent theta burst stimulation (TBS) based on an intracortical facilitation - (ICF) biomarker. A within-subjects linear mixed model analysis of repeated measures was conducted to assess changes in depressive symptoms using the Children’s Depression Rating Scale-Revised (CDRS-R). The analysis revealed a significant overall improvement in depressive symptoms from baseline to 6-month follow-up (p = 0.02; d=0.82). Significant pairwise comparisons were observed between baseline and week 2 [LSM decrease = −5.83 (SE = 2.`21), adjusted p = 0.04], and between baseline and 6-month follow-up [LSM decrease = −18.39 (SE = 7.05), adjusted p = 0.04]. There is a theoretical rationale for the use of ICF to guide dosing of TBS and this study suggests feasibility. While this study does not provide evidence to support the utility of the ICF biomarker, it may provide a template for future studies.

## Introduction

Major depressive disorder (MDD) is a leading causes of morbidity and mortality in adolescents, with a lifetime prevalence of approximately 11 % (Miller & Campo, 2021). At least 40 % of adolescents with MDD do not respond to evidence-based pharmacologic and psychotherapeutic treatments (Ayvaci & Croarkin, 2023). There are ongoing unanswered questions regarding dosing and sequencing TMS interventions in adolescents (Croarkin & MacMaster, 2019).

Theta Burst Stimulation (TBS) use has been increasing for adults with MDD since 2018 and research focused on adolescents is emerging (Dhami et al., 2019). Intermittent theta burst (iTBS) is thought to produce long-term potentiation-like effects, while continuous theta burst (cTBS) may be associated with long-term depression-like effects. As a result, cTBS may have cortical inhibitory effects, whereas iTBS may have cortical excitatory effects. (Huang et al., 2005).

One limitation of TBS treatment is the high variability of individual responses, influenced by factors that affect neuroplasticity, highlighting the need for personalized and targeted treatments (Corp et al., 2020). Recent research has focused on identifying the factors associated with this variability to better predict responses to TBS treatment (Katagiri et al., 2024; Lewis et al., 2024). Intracortical facilitation (ICF) has emerged as a promising biomarker of glutamatergic neurotransmission and synaptic plasticity. Measured via paired-pulse TMS of the motor cortex (M1), ICF serves as an index of glutamatergic N-methyl-D-aspartate (NMDA) receptor–mediated neurotransmission and overall cortical excitability due to shared neurophysiological mechanisms across cortical regions (Paulus et al., 2008; Ziemann et al., 2015). Recent evidence in adults suggests that reduced ICF, potentially driven by astrocytic dysfunction, contributes to treatment-resistant depression (Wada et al., 2024). Additionally, previous studies have demonstrated an association between ICF and depression severity in adolescents (Lewis et al., 2016), further supporting its relevance as a potential biomarker in this population. Baseline cortical excitability may also modulate TMS response (Cao et al., 2021; Farzan et al., 2013; Lewis et al., 2024). The motor threshold (MT) is an integral aspect of dosing TMS. Similar measures such as ICF may provide promising, scalable, and reliable measures of cortical excitability to guide treatment selection of cTBS versus iTBS for adolescent patients with depression.

With these considerations in mind, we propose that personalizing TBS treatment based on baseline ICF levels may enhance its effectiveness. In this exploratory study, adolescents who had not responded to standard 1 Hz and 10 Hz TMS treatments were offered a 2-week course of either cTBS or iTBS, stratified according to their baseline ICF measurements. We hypothesized that this biomarker-guided TBS approach would lead to improvements in both depressive symptoms and suicidality.

## Methods

### Study design

This exploratory study was the second phase of a larger study that has been published elsewhere (Lewis et al., 2024). This trial was prospectively registered (NCT03363919), and an investigational device exemption was obtained from the FDA (G170212). The study protocol was reviewed and approved by the Mayo Clinic Institutional Review Board (IRB) prior to the initiation of any research activities. Participants and their parents or guardians provided informed assent and consent before any study activities.

## Participants

The inclusion and exclusion criteria and the assessment of participants have been described in detail elsewhere (Lewis et al., 2024). Participants who did not have a response in depressive symptoms with standard 1 Hz or 10 Hz TMS were eligible (Lewis et al., 2024).

## Neurophysiology biomarker

Participants were assigned to treatment groups based on a paired-pulse TMS measure of ICF, a biomarker of NMDA glutamatergic-mediated cortical excitability, which was measured within 1 month prior to treatment. A higher ICF value reflects increased glutamatergic NDMA activity, and putative excesses in excitability and LTP-like synaptic plasticity. A lower ICF value reflects reduced glutamatergic NDMA activity with putative deficits in cortical excitability and overactive LTD-like synaptic plasticity. Participants with an ICF > 1.5 received cTBS for 2 weeks, whereas those with an ICF ≤ 1.5 received iTBS. The cutoff value was determined using data from a previous sample of seventy-one adolescents with depression, where this value represented a median split (Lewis et al., 2024.). ICF measurements with a fifteen millisecond interstimulus interval (ICF-15) were taken at baseline, week 1, and week 2. The fifteen millisecond interstimulus interval was selected based on prior work suggesting more clinical utility in adolescents with depression as compared to other inter-stimulus intervals (Lewis et al., 2024).

## Clinical assessments

Clinical assessments were conducted at baseline, week 1, week 2, and the 6-month follow-up by the principal investigator (P.E.C.) or trained clinical raters under supervision.

## Treatment protocol

TBS was applied to the left dorsolateral prefrontal cortex (LDLPFC) with the Beam F3 method based on an intent to examine TMS as commonly delivered in clinical practice. Participants receiving cTBS underwent 10 daily sessions (5 sessions per week for 2 weeks), with 120-second uninterrupted trains, delivering a total of 1800 pulses at 80 % MT (Li et al., 2014). Those receiving iTBS also underwent 10 daily sessions, with 2-s trains repeated every 10 s over a total duration of 570 s, delivering 1800 pulses at 80 % MT.

## Outcome variables

Depressive symptom severity (CDRS-R total score) assessed at baseline, week 1, week 2 and at 6-month follow-up was the primary outcome measure (Poznanski et al., 1984). The Columbian-Suicide Severity Rating Scale C-SSRS (Salvi, 2019) intensity of the ideation sub-scale, a secondary outcome, was measured at baseline, week 1, week 2, and at the 6-month follow-up. The ICF biomarker was measured at baseline, week 1, and week 2.

## Statistical analysis

Demographic and baseline clinical characteristics of the sample were described using the sample mean and standard deviation for continuous variables and the frequency and percentage for categorical variables. A completely within-subjects linear mixed model analysis of repeated measures was used to examine the change in depressive symptoms, suicidal ideation, and ICF-15, respectively, over the acute two weeks of TBS treatment as well as thru the 6-month follow-up period except ICF-15. A separate mixed model analysis was conducted on each of the outcomes. Restricted maximum likelihood estimation, Type 3 tests of fixed effects, and least squares were used, with the Kenward-Roger correction (Kenward & Roger, 1997) applied to the spatial power covariance structure. Changes in each outcome between baseline (pre-treatment) and each subsequent treatment period (weeks 1 and 2) and the 6-month follow-up were examined using least squares mean contrasts from the mixed model, and p-values associated with the tests of the mean contrasts were adjusted for multiple comparisons using the Dunnett procedure in SAS (SAS Institute, Inc., Cary, NC). Cohen’s *d* was calculated and interpreted as the effect size estimator for the omnibus within-subjects treatment effect.

We performed the statistical analyses using SAS software, version 9.4 (SAS Institute, Inc., Cary, NC). The procedures of PROC MIXED in SAS software were used to conduct the mixed model analysis. The level of significance was set at α = 0.05 (two-tailed) and to address multiple testing, p-values were adjusted using the False Discovery Rate (FDR) procedure.

## Results

### Participant characteristics

The study sample included 4 males (66.67 %) and 2 females (33.33 %), with an average age of 15.50 years (*SD* = 1.87, range = 12–17 years). Mean CDRS-R total, CSSRS intensity of ideation, and ICF-15 at baseline were 50.16 (*SD* = 8.13), 8.66 (SD = 9.50), and 1.35 (SD = 0.31), respectively. Participant characteristics are reported in Supplementary Table 1.

### Change in CDRS-R total over the acute TBS treatment and 6-month follow-up

**Fig. 1.**
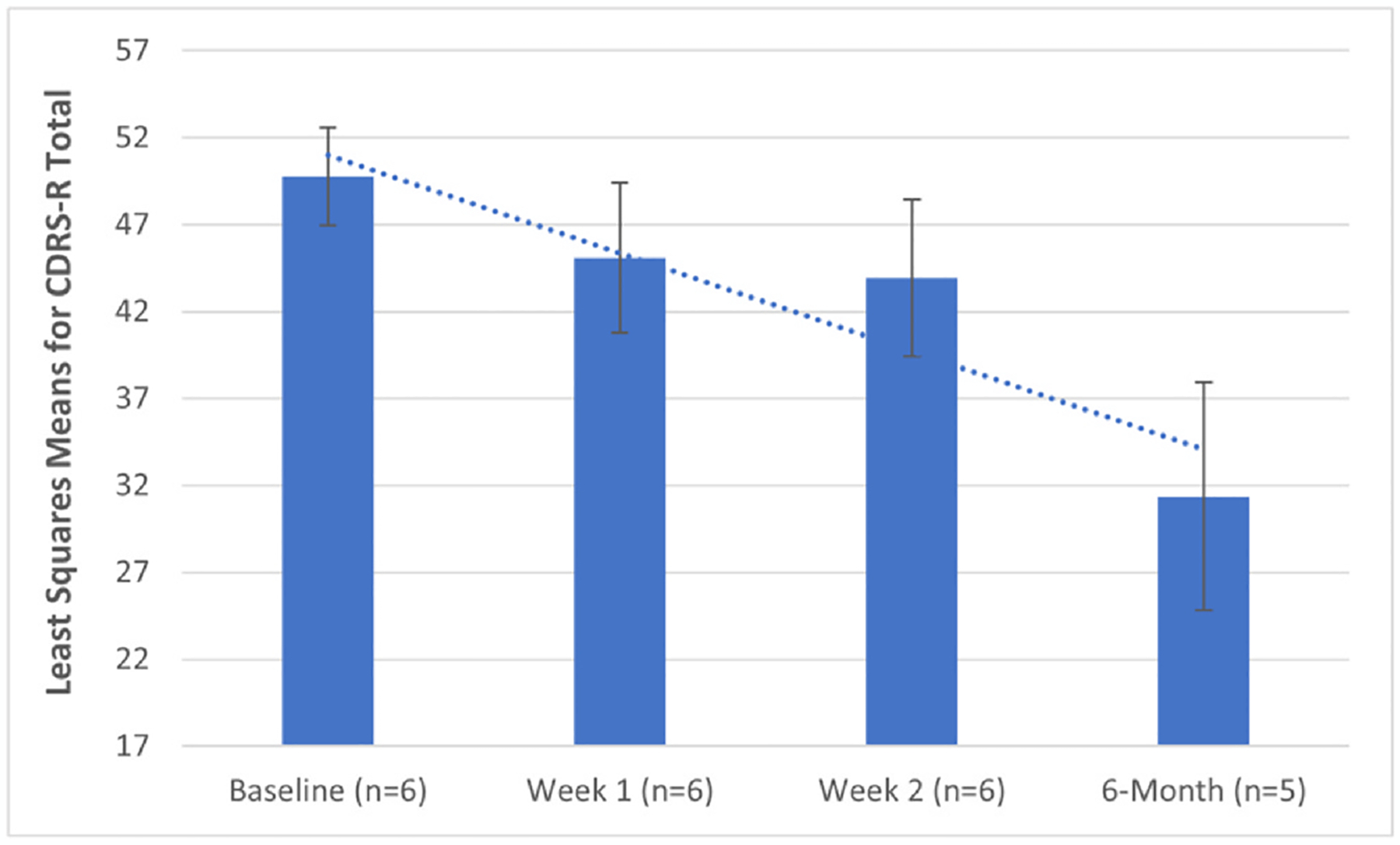
Adjusted least squares means (LSM ± SE) for CSSRS Intensity from the within-subjects linear mixed model for TBS treatment. LSM were adjusted for age. No overall significant change (decrease) was observed in CSSRS Intensity from baseline to the 6-month follow-up (p = 0.36). No significant pairwise comparisons were observed between baseline and any subsequent periods (adjusted p-values < 0.55).

**Fig. 2.**
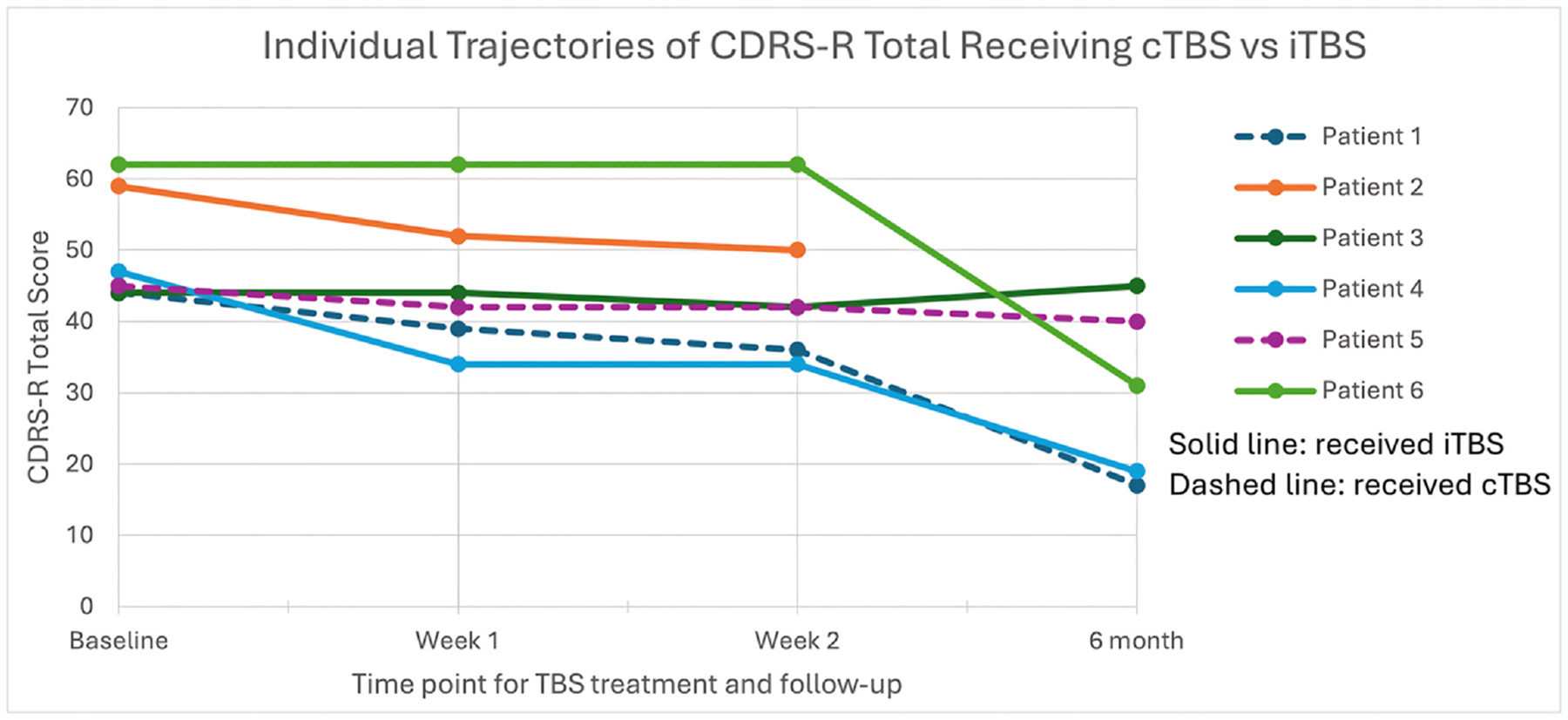
Individual trajectories of C-SSRS intensity of participants receiving cTBS or iTBS.

The results of the within-subjects linear mixed model analysis of repeated measures revealed an overall significant improvement (decrease) in CDRS-R Total from baseline to the 6-month follow-up (p = 0.0197; *d* = 0.82), with significant pairwise comparisons observed between baseline and week 2 [LSM decrease = −5.83 (SE = 2.21), adjusted p = 0.04] and baseline and 6-month follow-up [LSM decrease = −18.39 (SE = 7.05), adjusted p = 0.04]. Results are shown in Supplementary Table 2 and Fig. 1. The individual trajectories of CDRS-R scores are shown in Fig. 2.

### Change in CSSRS intensity over the acute TBS treatment and 6-month follow-up

The results of the within-subjects linear mixed model analysis of repeated measures revealed no overall significant change (decrease) in CSSRS Intensity from baseline to the 6-month follow-up (p = 0.36; *d* = 0.38; Supplementary Table 2 and Supplementary Fig. 2). No significant pairwise comparisons were observed between baseline and any subsequent periods (adjusted p-values < 0.55; Supplementary Fig. 1). The individual trajectories of CSSRS are shown in Supplementary Fig. 2.

### Change in ICF-15 over the acute TBS treatment

The results of the within-subjects linear mixed model analysis of repeated measures revealed no overall significant change (decrease) in ICF-15 from baseline to the week-2 (p = 0.49; *d* = 0.35; Supplementary Table 2). No significant pairwise comparisons were observed between baseline and any subsequent periods (adjusted p-values < 0.99; Supplementary Fig. 3). Individual trajectories of ICF-15 are shown in Supplementary Fig. 4.

## Discussion

This exploratory study examined the feasibility of a biomarker-guided TBS treatment in adolescents with moderate to severe MDD who previously did not respond to standard 1 Hz or 10 Hz TMS treatments. The results revealed improvements in depressive symptom severity after 2 weeks of treatment, with sustained benefits observed at the 6-month follow-up. While there was a trend toward reduced suicidal ideation, it did not reach statistical significance. There were no significant changes in ICF-15 levels over time, suggesting that ICF-15 may be a trait-like measure of NMDA receptor-mediated glutamatergic neurotransmission that remains stable during treatment (Lewis et al., 2024). This aligns with existing literature suggesting that ICF-15 may reflect baseline cortical excitability rather than being modulated by treatment (Chung et al., 2016; Lewis et al., 2024). ICF holds potential as a predictive marker for clinical outcomes. For example, Katagiri et al. demonstrated that ICF is the most important factor in predicting cTBS-induced synaptic plasticity in the lower limb motor cortex, supporting its role as a key biomarker for optimizing TBS protocols (Katagiri et al., 2024). Additionally, findings from the first course of this study indicated that participants in the low ICF group exhibited a better response to 1 Hz treatment compared to 10 Hz, further supporting its potential utility in guiding treatment selection (Lewis et al., 2024) However, it is important to note that the relationship between ICF and depression differs between adolescents and adults. While studies in adults have reported reduced ICF in depression (Wada et al., 2024), research in adolescents has found increased ICF in depressed youth (Croarkin et al., 2013). These findings highlight the developmental differences in excitatory-inhibitory balance, which remain incompletely understood. Given the vast neurophysiological differences between adolescents and adults, it is possible that age-specific mechanisms influence TMS response, necessitating different approaches for biomarker-guided interventions (Croarkin et al., 2013; Walther et al., 2009).

Treatment response to TBS in this feasibility study may reflect the potential impact of individualized variability based on ICF levels. While the present findings support the feasibility of using ICF to inform TBS treatment decisions in adolescents with depression, a larger study and replications are needed for definitive conclusion, whether ICF can serve as a definitive biomarker for predicting response and guiding treatment remains uncertain. It is possible that clinical improvements were independent of ICF stratification and reflect the general effectiveness of TBS in adolescents who had not responded to conventional 1 Hz and 10 Hz TMS, the delayed effects of treatment with 1 Hz or 10 Hz TMS, or simply the natural course of illness in this small sample of adolescents. To directly assess the role of ICF stratification in treatment outcomes, future studies should employ a larger, stratified design that compares treatment responses across groups of low and high ICF receiving either cTBS or iTBS.

This study has several limitations to note, and the findings should be viewed as speculative and qualitative. There are considerable threats to internal and external validity related to the small sample size and the conceptual approach (examining cTBS and iTBS in one experimental and statistical model). The absence of a control group, and the use of the Beam F3 method rather than neuro-navigation with magnetic resonance imaging are additional limitations. This present study suggests that ICF guided dosing of TBS in adolescent with depression is feasible, but it does not provide evidence for the utility or clinical effectiveness of ICF guided treatment. The present study may provide a template for future work with neurophysiologic biomarkers for personalizing TBS treatments, thereby offering a more targeted intervention which could enhance the treatment effectiveness while minimizing potential ineffective trials (Katagiri et al., 2020). Future randomized controlled trials with larger sample sizes are needed to validate these findings and to determine the most effective ways to personalize TBS interventions based on neurophysiological markers.

## Supplementary Material

2

1

3

4

5

6

Appendix A. Supporting information

Supplementary data associated with this article can be found in the online version at doi:10.1016/j.transm.2025.100093.
