# Supplementary material for: A feasibility study of sequenced TMS and TBS dosing in adolescents with major depressive disorder
Source: Transcranial Magn Stimul. Author manuscript; Available in PMC 2025 Jun 2. (PMC12128861; doi:10.1016/j.transm.2025.100093)
Supplement: 2 [file NIHMS2084094-supplement-2.docx]

| **Supplemental Table 2. Least Squares Means for clinical outcomes at Baseline and across the two weeks and followup for TBS Treatment** | | | | | | | | | | |  |
| --- | --- | --- | --- | --- | --- | --- | --- | --- | --- | --- | --- |
|  | Baseline | Week 1 | Week 2 | 6-Month Followup |  | Overall | | | | |  |
| Clinical Outcome | *LSM* (*SE*) n | *LSM* (*SE*) n | *LSM* (*SE*) n | *LSM* (*SE*) n | F Statistic | |  | p value | FDR | ES | |
| CDRS-R Total | 49.77 (2.83) 6 | 45.10 (4.32) 6 | 43.93 (4.50) 6 | 31.37 (6.54) 5 | F(3,14) = 4.57 | |  | 0.0197 | 0.05 91 | 0.82 | |
| CSSRS Intensity | 8.30 (3.98) 6 | 8.29 (3.97) 6 | 7.79 (3.70) 6 | 6.99 (3.12) 5 | F(3,14) = 1.09 | |  | 0.3633 | 0.4884 | 0.38 | |
| ICF15 | 1.35 (0.13) 6 | 1.65 (0.29) 6 | 1.37 (0.24) 6 | *No Follow-up* | F(2,10) = 0.77 | |  | 0.4884 | 0.4884 | 0.35 | |
|  |  |  |  |  |  | |  |  |  |  | |
| Note.  *LSM* = Least Squares Mean; *SE* = Standard Error; n = sample size; overall = baseline to week 2/follow-up | | | | | | | | | | | |
| F statistic was used to test for the omnibus (overall) mean change in clinical outcomes over time. | | | | | | | | | | |  |
| Adjusted least squares means (LSM±SE) for clinical outcomes from the within-subjects linear mixed model for TBS treatment. LSM were adjusted for age. | | | | | | | | | | |  |
| No 6-month followup for ICF15.  FDR = False Discovery Rate.  ES= Effect Size (Cohen’s d) | | | | | | | | | | |  |
