# Supplementary material for: A feasibility study of sequenced TMS and TBS dosing in adolescents with major depressive disorder
Source: Transcranial Magn Stimul. Author manuscript; Available in PMC 2025 Jun 2. (PMC12128861; doi:10.1016/j.transm.2025.100093)
Supplement: 1 [file NIHMS2084094-supplement-1.docx]

| **Supplementary Table 1. Participant Characteristics** | | |  |
| --- | --- | --- | --- |
|  |  |  |  |
|  |  |  |  |
|  |  | **cTBS group**  **(n=2)** | **iTBS group**  **(n=4)** |
| **Participant Characteristic** |  |  |  |
|  |  |  |  |
| **Demographics** |  |  |  |
| Age in years, M (SD) |  | 16.5 (0.71) | 15 (2.16) |
| Sex, n |  |  |  |
| Male |  | 0 | 4 |
| Female |  | 2 | 0 |
| **Clinical Characteristics** |  |  |  |
| CDRS-R total at baseline, M (SD) |  | 44.5 (0.5) | 54 (8.83) |
| CSSRS Intensity at baseline, M (SD) |  | 9 (12.73) | 8.5 (9.81) |
| ICF15 at baseline, M (SD) |  | 1.70 (0.25) | 1.18 (0.14) |
|  |  |  |  |
| Note. CDRS-R = Children’s Depression Rating Scale Revised; CSSRS = Columbia Suicide Severity Rating Scale; ICF-15 = Intracortical Facilitation with 15 millisecond interstimulus interval; M = Mean; SD = Standard Deviation; N = sample size at baseline. | | |  |
