# Supplementary figures and images for: A feasibility study of sequenced TMS and TBS dosing in adolescents with major depressive disorder
Source: Transcranial Magn Stimul. Author manuscript; Available in PMC 2025 Jun 2. (PMC12128861; doi:10.1016/j.transm.2025.100093)

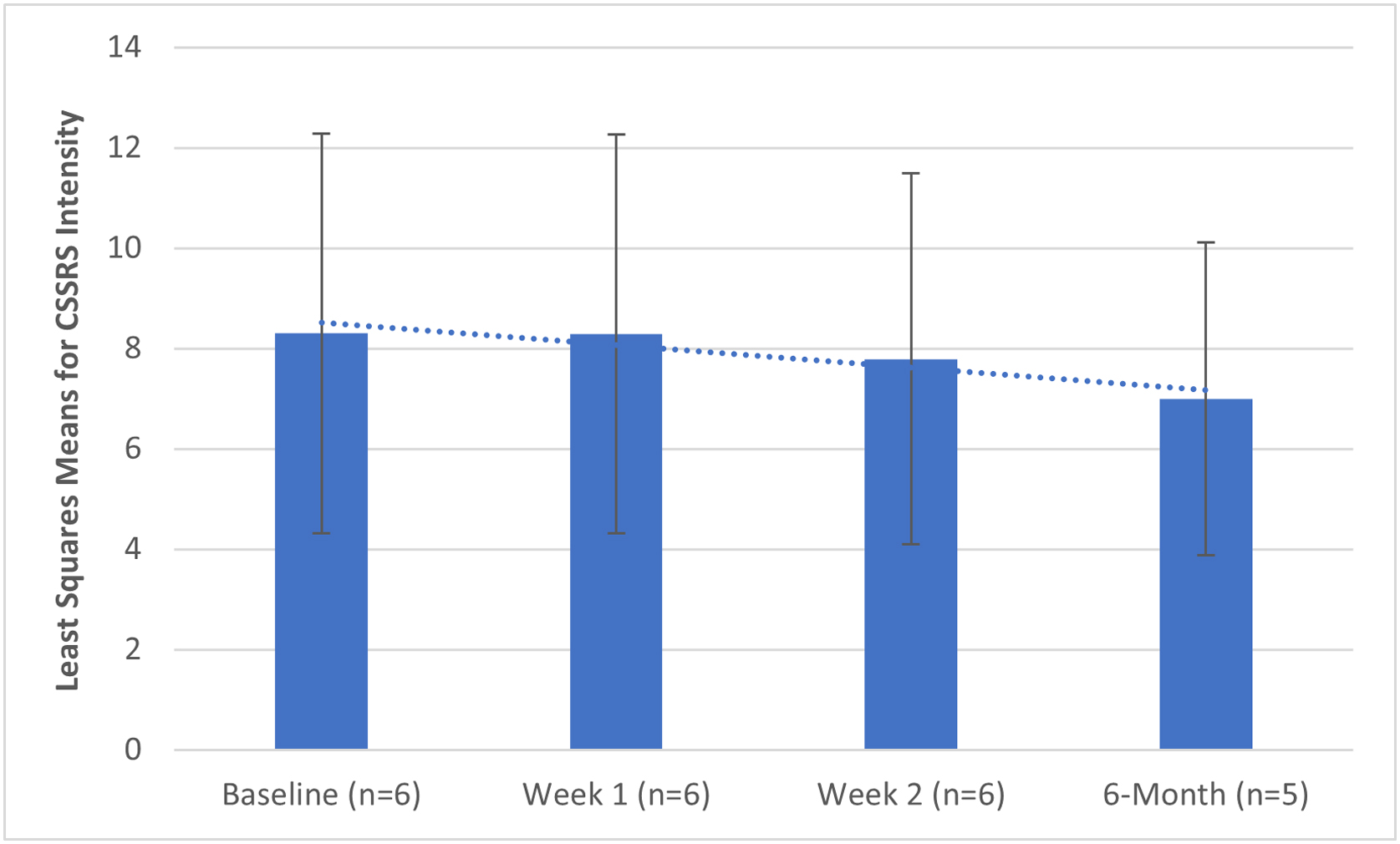

Supplement: 3 [file NIHMS2084094-supplement-3.jpg]

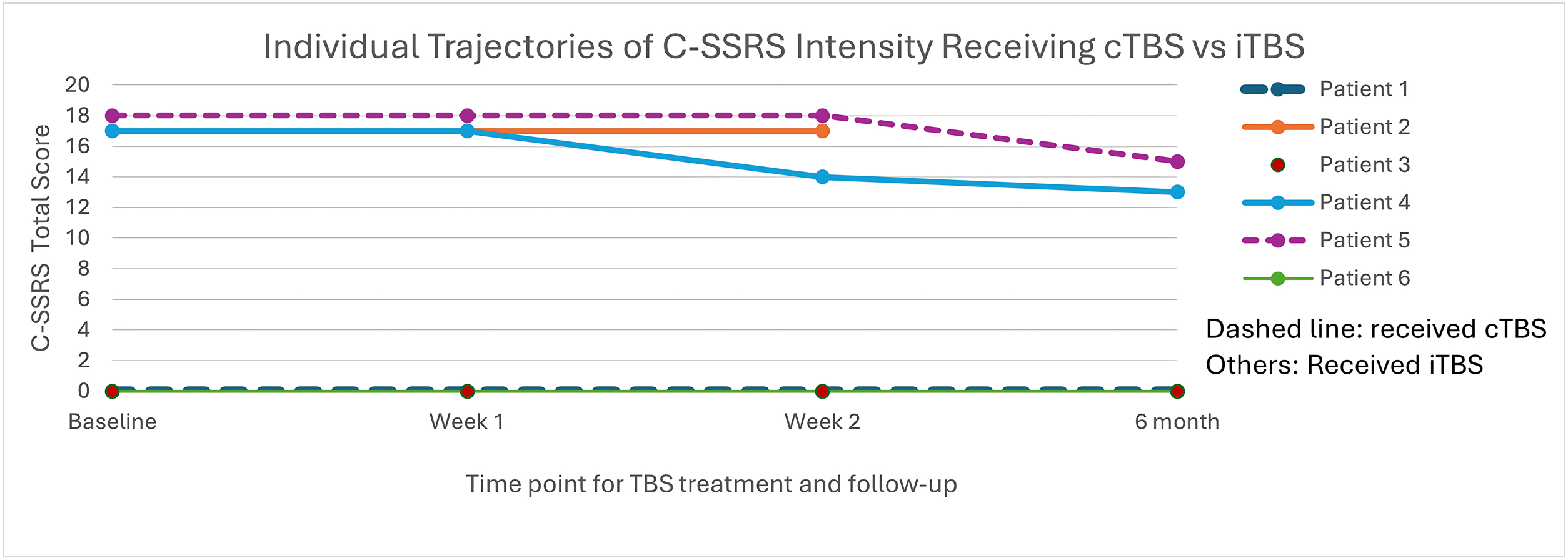

Supplement: 4 [file NIHMS2084094-supplement-4.jpg]

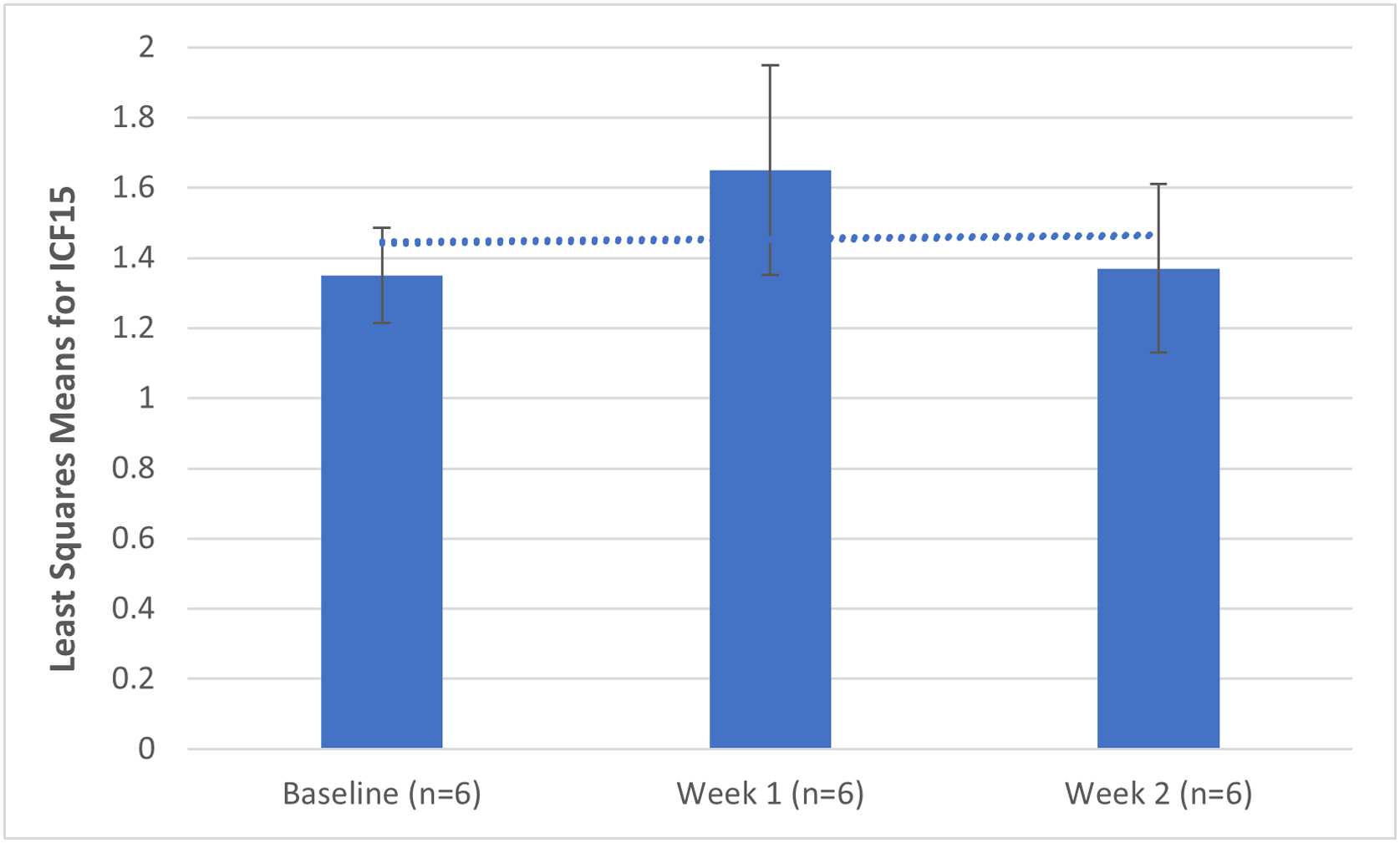

Supplement: 5 [file NIHMS2084094-supplement-5.jpg]

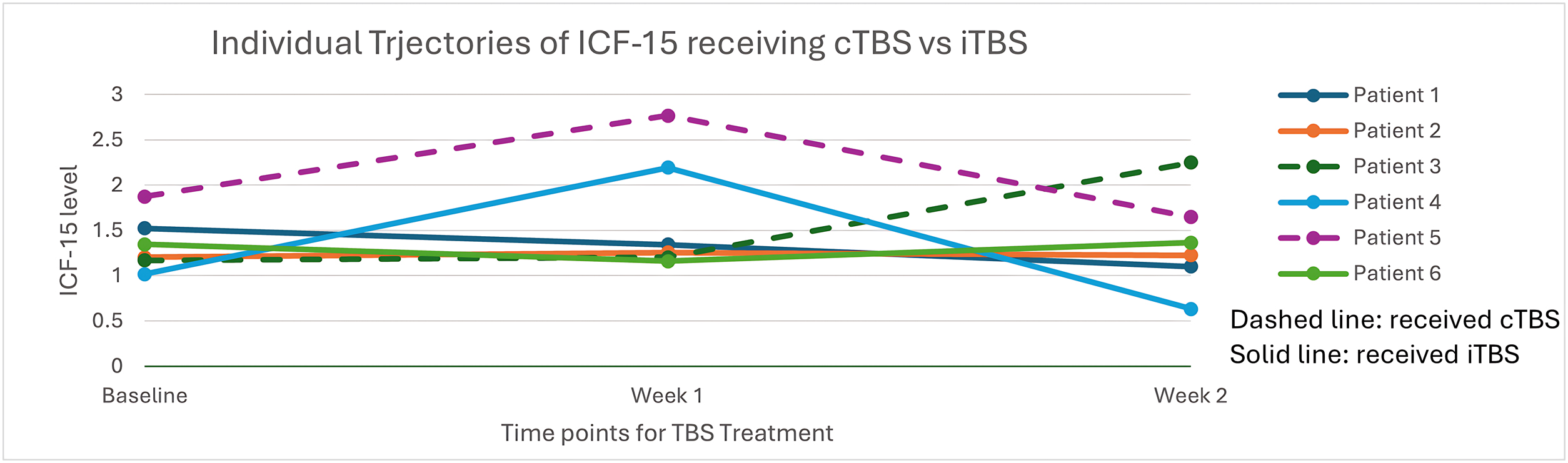

Supplement: 6 [file NIHMS2084094-supplement-6.jpg]
